# Recruitment strategies for predominantly low-income, multi-racial/ethnic children and parents to 3-year community-based intervention trials: Childhood Obesity Prevention and Treatment Research (COPTR) Consortium

**DOI:** 10.1186/s13063-019-3418-0

**Published:** 2019-05-28

**Authors:** Zhaohui Cui, Kimberly P. Truesdale, Thomas N. Robinson, Victoria Pemberton, Simone A. French, Juan Escarfuller, Terri L. Casey, Anne M. Hotop, Donna Matheson, Charlotte A. Pratt, Lynn J. Lotas, Eli Po’e, Sharon Andrisin, Dianne S. Ward

**Affiliations:** 1 Department of Nutrition, Gillings School of Global Public Health, University of North Carolina at Chapel Hill, Chapel Hill, NC USA; 2 Stanford Solutions Science Lab, Department of Pediatrics, Stanford University School of Medicine, Stanford, CA USA; 3 National Institutes of Health, National Heart, Lung, and Blood Institute, Bethesda, MD USA; 4 Division of Epidemiology & Community Health, School of Public Health, University of Minnesota, Minneapolis, MN USA; 5 Department of Pediatrics, Vanderbilt University Medical Center, Nashville, TN USA; 6 Rainbow Babies & Children’s Hospital, Cleveland, OH USA; 7 Frances Payne Bolton School of Nursing, Case Western Reserve University, Cleveland, OH USA

**Keywords:** Recruitment, Strategy, Barrier, Intervention, Minority, Hispanic, African American, Low-income, Children, Parent–child dyads

## Abstract

**Background:**

The recruitment of participants into community-based randomized controlled trials studying childhood obesity is often challenging, especially from low-income racial/ethnical minorities and when long-term participant commitments are required. This paper describes strategies used to recruit and enroll predominately low-income racial/ethnic minority parents and children into the Childhood Obesity Prevention and Treatment Research (COPTR) consortium.

**Methods:**

The COPTR consortium has run four independent 3-year, multi-level (individual, family, school, clinic, and community) community-based randomized controlled trials. Two were prevention trials in preschool children and the other two were treatment trials in pre-adolescents and adolescent youth. All trials reported monthly participant recruitment numbers using a standardized method over the projected 18–24 months of recruitment. After randomization of participants was completed, recruitment staff and investigators from each trial retrospectively completed a survey of recruitment strategies and their perceived top three recruitment strategies and barriers.

**Results:**

Recruitment was completed in 15–21 months across trials, enrolling a total of 1745 parent-child dyads- out of 6314 screened. The number of children screened per randomized child was 4.6 and 3.5 in the two prevention trials, and 3.1 and 2.5 in the two treatment trials. Recruitment strategies reported included: (1) careful planning, (2) working with trusting community partners, (3) hiring recruitment staff who were culturally sensitive, personality appropriate, and willing to work flexible hours, (4) contacting potential participants actively and repeatedly, (5) recruiting at times and locations convenient for participants, (6) providing incentives to participants to complete baseline measures, (7) using a tracking database, (8) evaluating whether participants understand the activities and expectations of the study, and (9) assessing participants’ motivation for participating. Working with community partners, hiring culturally sensitive staff, and contacting potential participants repeatedly were cited by two trials among their top three strategies. The requirement of a 3-year commitment to the trial was cited by two trials to be among the top three recruitment barriers.

**Conclusions:**

Comprehensive strategies that include community partnership support, culturally sensitive recruitment staff, and repeated contacts with potential participants can result in successful recruitment of low-income racial/ethnic minority families into obesity prevention and treatment trials.

**Trial registration:**

NET-Works trial: ClinicalTrials.gov, NCT01606891. Registered on 28 May 2012.

GROW trial: ClinicalTrials.gov, NCT01316653. Registered on 16 March 2011.

GOALS trial: ClinicalTrials.gov, NCT01642836. Registered on 17 July 2012.

IMPACT trial: ClinicalTrials.gov, NCT01514279. Registered on 23 January 2012.

**Electronic supplementary material:**

The online version of this article (10.1186/s13063-019-3418-0) contains supplementary material, which is available to authorized users.

## Background

The recruitment of study participants into community-based interventions is the foundation for a successful trial. Contemporary community-based interventions often focus on economically disadvantaged, racially/ethnically diverse populations [1]. Recruitment of these populations requires an understanding of the challenges faced by potential participants, such as feelings of mistrust of medical care and medical research [1–4], family stressors including high mobility and financial distress [5], lack of a working phone to communicate with research staff [5], inflexible work hours or lack of time due to working several jobs [5], lack of childcare or transportation [6], low literacy skills [5], and limited understanding of research purposes and processes [1]. When intervention studies require a long-term commitment (e.g., more than a year), the recruitment of participants is more challenging than for a short-term study [7].

Childhood obesity has been associated with hypertension [8], cardiometabolic abnormalities [8], cardiovascular dysfunction [9], sleep disorders [8], psychological difficulties [10, 11], and adult obesity [12]. The total direct medical costs of childhood obesity have been estimated at $14.1 billion annually [13]. Childhood obesity is an important public health problem, and is up to three times more prevalent among low-income, racial/ethnic minority children [14]. Despite its importance, our recent systematic review of published pediatric obesity trials registered in the Clinical Trials Registration database identified little information on recruitment of low-income minority families into long-term obesity prevention or treatment trials [7]. One reason may be that parents may not perceive their child to be at risk of obesity [15]. Health-care providers are also unlikely to diagnose obesity in children [16, 17]. Even when diagnosed, parents, especially low-income parents, may not prioritize obesity as an important health problem for their children, given a host of more immediate stressors and challenges they may face [18, 19].

The Childhood Obesity Prevention and Treatment Research (COPTR) consortium is funded by the National Institutes of Health (NIH) to develop and evaluate four distinct multi-level (individual, family, school, clinic, and community) and multi-component community-based child obesity prevention and treatment trials, including individualized recruitment protocols. The COPTR consortium collaborated to share, refine, and document recruitment strategies systematically and successfully enrolled 1745 low-income racially/ethnically diverse parent–child dyads. A retrospective reflection of the COPTR recruitment experience offers a unique opportunity to identify strategies that resulted in successful recruitment in this hard-to-enroll population.

This paper describes the strategies used to recruit and enroll predominately low-income racial/ethnic minority parents and children into the four community-based obesity prevention and treatment trials run by the COPTR consortium. It may provide insight for future investigators seeking to enroll similar populations.

## Methods

The COPTR consortium ran for four independent community-based randomized controlled trials and a research coordinating unit (RCU) funded by the NIH [20]. The study designs have been previously described [20–24]. The two prevention trials targeted preschool children and were conducted by the University of Minnesota [21] and Vanderbilt University [22], respectively. The two treatment trials targeted pre-adolescents and were conducted by Case Western Reserve University (CWRU) [23] and Stanford University [24], respectively. All four trials were 3-year multi-level interventions and enrolled low-income racial/ethnic minority populations. The four trials had their own recruitment methods, interventions, and measurement protocols and agreed a priori to collect an extensive array of common measures [20]. To reach a low-income population, one trial (Minnesota) used annual family income below $65,000 [21] as an eligibility criterion, while the other trials recruited participants in low-income neighborhoods and did not apply an income criterion [22–24]. By design and based on the power calculations for each trial, recruitment goals varied for the prevention (*n* = 500 and 600) and the treatment (*n* = 240 and 360) trials [21–24].

The RCU was located at the University of North Carolina at Chapel Hill. The RCU supported the development of data collection protocols for common measures and coordinated the collaborative working committees, subcommittees, and working groups, and interactions with the consortium’s NIH-appointed independent data and safety monitoring board. The consortium-wide Recruitment, Consent, Retention and Adverse Events (RCRAE) subcommittee consisted of a staff member responsible for recruitment and an investigator from each trial. The RCRAE subcommittee was responsible for the development of a cross-trial standardized method to report recruitment progress over the consortium’s 18-month recruitment phase (24 months for Vanderbilt). Each trial reported monthly recruitment and enrollment progress to the RCRAE subcommittee using four predefined phases: screening, eligibility, consent, and randomization. Reasons for discontinuation during the recruitment process were documented. Recruitment progress was reviewed monthly by the RCRAE subcommittee. The monthly RCRAE meetings provided opportunities for the committee members to discuss perceived effective strategies, lessons learned, and solutions to recruitment barriers. The site representatives further discussed those solutions within their sites and decided whether and how to implement those potential solutions. The RCU prepared and submitted quarterly progress reports to the NIH and biannual progress reports to the data and safety monitoring board for review.

**Fig. 1:**
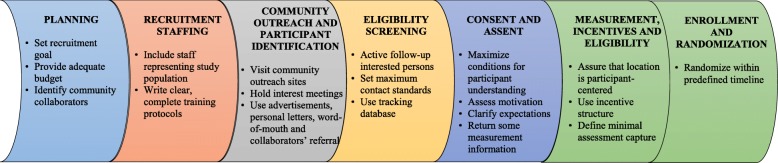
Seven stages and key components of recruitment

Two and a half years after the randomization of participants across all four trials was completed, the RCU created a list of the key recruitment strategies frequently documented in the literature based on our recent systematic review [7] and categorized them into seven stages primarily based on temporal sequence: (1) planning, (2) recruitment staffing, (3) community outreach and participant identification, (4) eligibility screening, (5) consent and assent, (6) measurement, incentives, and eligibility, and (7) enrollment and randomization (Fig. 1). Based on these seven stages of recruitment, the RCU developed a survey with open-ended questions that asked each trial group what recruitment strategies they had used (Additional file 1: Table S1). This retrospective survey also included open-ended questions to query trial groups about their perceived top three recruitment strategies and barriers. In addition, each trial group identified barriers they had experienced from a list developed by the RCU based on a literature review. The survey questions were sent to the staff member in each trial who was responsible for recruitment and who had served on the RCRAE subcommittee. Each site independently completed the survey questions with input from the recruitment staff member and the investigator. One completed survey from each trial was sent to the RCU and the RCU manually coded and summarized the responses.

## Results

The four COPTR trials successfully recruited their targeted number of children within their projected recruitment phases. All four trials retrospectively reported using similar and different recruitment strategies to achieve their recruitment goal (Table 1).

**Table 1:** Recruitment strategies used by each trial

	Minnesota^a^ (*n* = 534)	Vanderbilt^a^ (*n* = 610)	Stanford^b^ (*n* = 241)	CWRU^b^ (*n* = 360)
*Planning*				
Created recruitment plan	X	X	X	X
Rolling recruitment	X	X	X	
Created community advisory boards	X	X	X	X
Pilot test recruitment strategy	X	X	X	X
*Recruitment staffing*				
Full-time bilingual staff (English/Spanish)	X	X	X	
Outreach staff for African American communities		X		X
Recruitment staff from community	X	X	X	X
Written recruitment protocol	X	X	X	X
*Community outreach and participant identification*				
Community outreach	X	X	X	X
Partnered with primary care clinics	X			
Partnered with local school system				X
Direct community-based strategies		X	X	
Held interest meetings		X	X	X
Media advertisement		X	X	X
*Eligibility screening*				
Followed recruitment protocol	X	X	X	X
Set limits on the number of call attempts	X	X	X	X
Used tracking software for recruitment	X	X	X	X
Assessed participation motivation	X			
*Consent and assent*				
Provided clear and easy consent and assent materials	X	X	X	X
Consent materials at 4th – 7th grade reading level	X	X	X	X
Had an outside group review the consent and assent materials	X	X	X	X
Certificate of confidentiality or discussion of patient privacy	X	X	X	X
*Measurement, incentives, and eligibility*				
Protocol for scheduling and re-scheduling visits	X	X	X	X
Baseline data collection at family’s home	X			
Baseline data collection at local community center		X	X	
Baseline data collection at university-based clinical research center			X	X
Minimum baseline data requirement	X	X	X	X
Monetary incentive provided	X	X	X	X
Reimbursed for transportation or parking		X		X
Provided participants with laboratory results			X	X
*Enrollment and randomization*				
Randomized participants within 37 days of height and weight measurements	X	X	X	X

^a^Prevention trial

^b^Treatment trial

### Recruitment strategies

#### Planning

Each trial group produced a recruitment plan before their study started. The COPTR consortium leveraged the collective experience of the teams and held monthly calls to plan recruitment in a variety of recruitment settings (primary care clinics, schools, churches, community centers, libraries, local businesses, and community events). Rolling recruitment was planned for three trials (Minnesota, Vanderbilt, and Stanford), while CWRU recruited in two separate cohorts timed with the school year. All four trials created community advisory boards or had assistance from community partners with recruitment activities. Advanced planning permitted sites to allocate financial resources to recruitment activities.

#### Recruitment staffing

Stanford and Vanderbilt aimed to recruit primarily Hispanic/Latino children. CWRU and Minnesota aimed to recruit racially/ethnically diverse children. Three trials employed full-time bilingual staff (English/Spanish) for recruitment, data collection, and intervention activities. CWRU and Vanderbilt also employed outreach staff to work with African American communities. Some recruitment staff lived in the communities. Each trial used a written recruitment protocol to train recruitment staff. The protocols included screening, recruitment, and consent and assent processes.

#### Community outreach and participant identification

Community outreach was used by all trials to generate potential participant interest. Minnesota partnered with three primary care clinic systems (a total of 12 primary care clinics) and used electronic medical records to identify eligible children due to their age and body mass index (BMI). CWRU partnered with the Cleveland Public School District to identify children through a district-wide BMI screening. Vanderbilt and Stanford used direct community-based strategies that included in-person recruitment at schools, churches, grocery stores, and neighborhood recreational centers. Interest meetings were held by three of the four trials (Vanderbilt, Stanford, and CWRU). These same trials also used posters, flyers, school banners, personalized recruitment letters, word-of-mouth, and radio or television announcements to promote study participation.

#### Eligibility screening

Interested participants were contacted directly by recruitment staff. Recruitment phone protocols were used by all four trials and included a maximum number of call attempts in a given period (e.g., five calls within 30 days) with instructions about leaving messages, consecutive hang-ups, and handling disconnected numbers. Policies were implemented at some trials to re-contact potential participants after consecutive failures to ensure they attended their scheduled enrollment visits. Tracking software (e.g., REDCap and FileMaker Pro) or other software (e.g., Google Documents) was commonly used. Software can help recruitment staff to understand the progress of recruitment, and to plan and remind them of future recruitment activities. Although less structured, efforts were made by one trial (Minnesota) to assess participation motivation to ensure participant interest prior to enrollment.

#### Consent and assent

A high priority for all trials was to ensure that the consent and assent processes were clear and easy for participants to understand. The proportion of caregivers with an education level less than high school graduate ranged from 20% to 60%. The reading levels of the consent materials were carefully considered and ranged from 4th to 7th grade. Sites used a variety of methods, including having a group of parents like the study population preview the consent materials, employing simple questions at the end of the consent process to assess potential participants’ understanding, or using visual aids (e.g., pictures used by Minnesota, Additional file 2: Figure S1 and Additional file 3: Figure S2). All trials used strategies to ensure that participants understood the benefits and potential risks of study participation. A certificate of confidentiality or an explicit discussion of the privacy of participant information were deemed particularly important in these trials, since a large proportion of the families may have been recent immigrants.

#### Measurement, incentives, and eligibility

Each trial followed its own protocol for scheduling and re-scheduling missed or cancelled visits, for specific measures (such as dietary recalls and accelerometry), and for the number of data collection attempts. Baseline data collection occurred at the family’s home (Minnesota), a local community center (Vanderbilt and Stanford), or a university-based clinical research center (CWRU and Stanford). The COPTR consortium required a minimum set of baseline data to be collected before a participant could be randomized. These included measured height and weight, common demographic information, a minimum of two 24-h dietary recalls (Stanford required three), a minimum of three weekdays and one weekend day of accelerometer wear data with at least 6 hours of useable data each day, and fasting blood samples (Stanford and CWRU). Participants were required to complete all baseline measures within a 30-day window. Recruitment staff were available to answer questions from participants and remind them about data collection appointments.

Monetary incentives for completion of data collection (total value ranged from $40 to $110) were offered and distributed in a variety of ways. Trials either provided a reimbursement to families after specific segments of data collection were completed, or waited until all data had been collected. Minnesota gave a $10 gift card at each of two home visits and a $30 gift card after completion of the third dietary recall and accelerometer wear. Vanderbilt distributed a $20 gift card on the day of data collection and another $20 gift card upon return of the accelerometer to research staff. CWRU distributed a $50 gift card to each parent and each child on the day of data collection and another $10 gift card to the child upon return of the accelerometer to research staff. Stanford gave a $50 gift card after the completion of baseline measurements. Transportation or parking reimbursement, childcare and activities to keep siblings occupied while working with the adult participants, and small token gifts augmented the monetary incentives. Two trials (Stanford and CWRU) offered enrolled participants a subset of the initial examination results (e.g., lipid profile, fasting glucose level, and blood pressure).

#### Enrollment and randomization

The COPTR trials randomized participants within 37 days of their initial height and weight measurements. The mean (standard deviation) number of days between baseline weight and height measurements and randomization were 19.6 (6.1) at Minnesota, 20.7 (12.4) at Vanderbilt, 15.0 (4.7) at Stanford, and 22.6 (6.7) at CWRU.

### Recruitment outputs

**Fig. 2:**
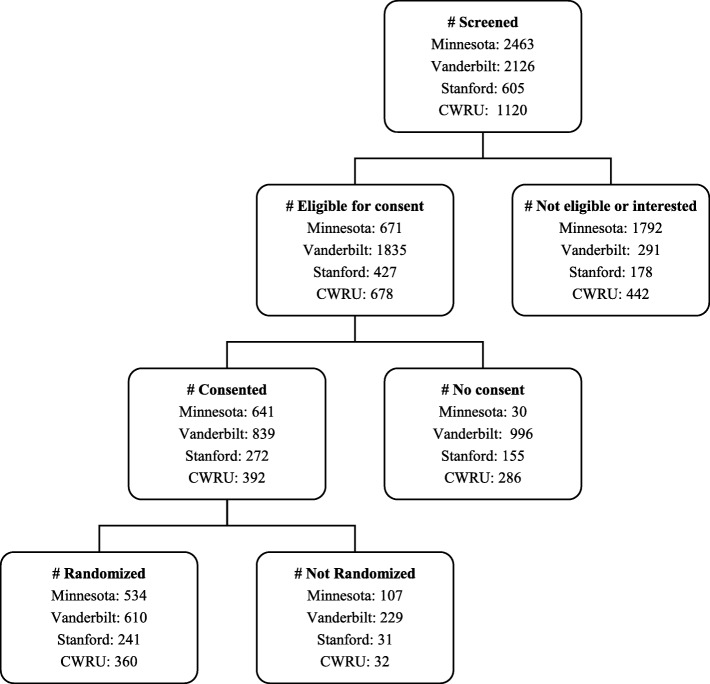
Consort diagram of recruitment of parent-child dyads by trial. CWRU Case Western Reserve University

Figure 2 shows the number of potential subjects at each phase (screened, eligible, consent completed, and randomized) for each trial. Across trials, recruitment was completed in 15–21 months. All four trials achieved or exceeded their recruitment goals. Minnesota had a goal of recruiting 500 dyads and enrolled 534; Vanderbilt aimed to recruit 600 and enrolled 610; Stanford aimed to recruit 240 and enrolled 241, while CWRU aimed to recruit and enrolled 360. The number of children screened per randomized child was 4.6, 3.5, 2.5, and 3.1 at Minnesota, Vanderbilt, Stanford, and CWRU, respectively. Additional file 4: Table S2 lists reasons for exclusion of potential participants from the study. Additional file 5: Table S3 shows the baseline characteristics of the randomized COPTR participants by trial.

### Recruitment strategies and barriers

Table 2 displays the recruitment staff and investigators’ perceived top three strategies and barriers. The four trial groups reported a total of nine recruitment strategies that they perceived to be effective: (1) flexible work schedules allowing staff to meet participants outside regular work hours, (2) clear detailed recruitment protocols, (3) repeated contacts, (4) existing trustful relationship with the community, (5) use of community liaison, (6) a tracking database, (7) culturally sensitive staff, (8) face-to-face recruitment, and (9) personal style of staff. Trusting relationship, repeated contacts, and culturally sensitive staff were cited by two trials as among their top three strategies. The other strategies were mentioned by only one trial each. The four trials reported eleven key barriers to recruitment: (1) wearing the accelerometer for 7 days, (2) participants’ loss of interest, (3) participants’ lack of understanding or knowledge of the research, (4) busy and unpredictable family schedules, (5) lack of transportation, (6) narrow BMI eligibility criteria, (7) cultures that did not value obesity prevention, (8) the 3-year commitment to the trial, (9) the unavailability of a roster from which to select potential participants, (10) inaccurate phone numbers, and (11) an inability to reach out to a caregiver in each household. The 3-year commitment to the trial was cited by two trials as among their top three recruitment barriers.

**Table 2:** Perceived top three recruitment strategies and barriers reported by each trial

Minnesota (*n* = 534)	Vanderbilt (*n* = 610)	Stanford (*n* = 241)	CWRU (*n* = 360)
*Top three strategies*			
Staff working hours to meet participants needs (morning, afternoon, and evening)	Building trusted relationships in our community over the prior 5 years and soliciting input from trusted community leaders to guide our processes from the outset	Staff who are culturally competent and able to communicate the requirements of the research study in language accessible to our sample	Long-term working relationship with school (nurses); families trust their schools
Clear, detailed protocols allowed for systematic recruitment	Used the community liaison model. Essentially, leveraging trust and trusting relationships	Face-to-face recruitment, and actively approaching potential participants in their community	Staff were well trained and diligent
Repeated contacts	Creating a tracking database to identify real-time staffing needs and return on investment	Multiple contacts with families to ensure that they understand the expectations of the trial, maintain interest, and are committed to participating in the research	Personal style of recruitment staff (warm, friendly, and professional)
*Top three barriers*			
Accelerometer wear time requirements	Not valuing prevention, since their child was well and they wanted to avoid the stigma of being labelled “unwell”	Family schedules that are unpredictable and very busy	Accurate phone numbers
Loss of interest between home visits 1 and 2	The level of commitment over 3 years seemed burdensome and unrealistic, and not wanting to lose face by dropping out later	Lack of reliable transportation for some	Length of study, 3 years
Lack of understanding or knowledge of the research	Eligibility included BMI over 50% but not yet obese; this narrow eligibility requirement meant it took much longer to recruit than would have been the case with our originally proposed criteria, of which we had prior experience and success	Finding eligible families in our community setting without having a list of potentially eligible patients or school class lists of names and contact information	Reaching a parent or guardian in each household

*BMI* body mass index

Table 3 lists 21 items that were frequently cited in the existing literature as recruitment barriers, and responses from the four COPTR trials about whether those barriers existed for their own trial. Only four of the 21 items from the broader literature were considered to be barriers by the majority (three or four) of COPTR trials: (1) time demands and scheduling conflicts, (2) disconnected phone numbers, (3) transportation to research site, and (4) data collection completeness requirement. Eleven of the 21 barriers were endorsed by one or two COPTR trials. Six barriers were not endorsed by any of the trials: (1) Community collaborators unfamiliar with study, (2) extra paperwork for the participants, (3) low level of literacy or numeracy, (4) concerns that not all familial members would benefit, (5) failure to describe the study accurately, and (6) inability to track the progress of potential participants.

**Table 3:** Perceived recruitment barriers given in the literature as reported by each trial

Description	Minnesota (*n* = 534)	Vanderbilt (*n* = 610)	Stanford (*n* = 241)	CWRU (*n* = 360)
Time demands and scheduling conflicts	X	X	X	X
Disconnected phone number	X	X	X	X
Transportation to research site		X	X	X
Data collection requirement	X	X	X	
Challenge working with a large group of institutions or organizations	X			X
Limited e-mail access	X			X
Transient population	X			X
Participants unfamiliar with research and study participation	X			X
Mails sent from school not received by family	X			X
Lack of interest	X			X
Feeling of mistrust	X			
No staff from study population	X			
Families failure to initiate interest in study			X	
Limited number of bilingual staff				X
Needing both parent and child participation				X
Community collaborators unfamiliar with study				
Extra paperwork for the participants				
Low level of literacy or numeracy				
Familial concerns that not all familial members will benefit				
Failure to describe the study accurately				
Inability to track the progress of potential participants				

## Discussion

The COPTR consortium successfully recruited 1745 low-income parent-child dyads into two prevention and two treatment trials for child obesity. Recruitment strategies from the four trials were categorized into seven stages based on a comprehensive review of the recruitment literature. Although recruitment strategies used in trials and cohort studies have been previously published, those work has not defined and classified the recruitment process into specific stages. These stages provide a step-by-step framework that may help investigators better understand the demands on recruitment resources and allow them to plan and implement strategies for successful recruitment more efficiently.

Incentives are an important, yet less discussed, element of successful participant recruitment and may be particularly relevant when recruiting lower-income study participants [7]. Incentives that are offered for data collection in child obesity prevention and treatment studies include a variety of forms such as gifts, food, recipe books, exercise equipment, grocery gift cards, and cash [7]. Among these, a monetary incentive is one of the most commonly used. Monetary incentives can enhance data collection response rates, but must be balanced with the cost per participant enrolled [25] and the potential for coercion. Of the 43 studies from our systematic review [7], we were able to identify only one child obesity intervention study conducted in an underserved population that reported the use of monetary incentives. In a trial to prevent weight gain in Hispanic children aged 2–6 years, a total value of approximately $30 in incentives (e.g., a cutting board, a kitchen timer, and a gift card for a local supermarket) were distributed to participants after collecting all the baseline data [26]. In the present four COPTR consortium trials, gift cards totaling $40, $50, or $110 were perceived to be effective incentives to compensate families for the time and effort required to complete baseline recruitment and to produce the observed high measurement completion rates among participants in geographically and racially/ethnically diverse low-income families. Whilst, different incentive schedules could have different effects on participant recruitment, the four trials in our study did apply different incentive schedules and all trials successfully recruited participants.

A unique feature of the COPTR consortium is that it runs both prevention and treatment trials. This enabled a rough comparison of the recruitment of participants into childhood obesity prevention versus treatment trials. The number of screened children per randomized child in the two prevention trials was larger than that in the two treatment trials. This is consistent with our systematic review [7]. There are several speculative explanations. One is that prevention trials included children in the normal BMI percentile range; therefore, their parents may not be as concerned about childhood obesity as the parents of overweight or obese children. Furthermore, children with a BMI lower than the 50th percentile were not eligible for the COPTR prevention trials, narrowing the eligible pool. It can be difficult for recruitment staff to exclude those children accurately without measuring them, potentially increasing the ratio of children screened to randomized.

Collecting all baseline data before randomization has been widely used in the implementation of randomized controlled trials to enhance data completion and retention rates [27–29]. The COPTR consortium required a minimum set of baseline data to be collected for a family before it was eligible for enrollment and randomization. Collecting baseline data helps participants understand the burden of data collection and serves as the entry point into an intervention study. Those who do not provide the minimum data at baseline may be more likely to provide insufficient data or drop out during the follow-up. Having many missing values may introduce bias and erode the statistical power. Although the minimum baseline data requirement could reduce the generalizability by excluding participants who showed less adherence to the recruitment process, the COPTR consortium investigators decided that balancing these potential threats favored requiring a minimum set of baseline data. In an effort to reduce loss to follow-up, recruitment staff clearly communicated study demands and researchers’ expectations of participation at the screening and consent stages to ensure that participants accurately understood the benefits, potential risks, and time commitments of the interventions and assessments. Some investigators have suggested that before potential participants are enrolled into a study, their willingness to participate should be evaluated. Those who seem hesitant to participate or cooperate should not be enrolled [30].

Successful recruitment into the four trials of the COPTR consortium may also be partially attributed to its organization and structure. The regular conference calls within the RCRAE subcommittee and the biannual review of progress with the data and safety monitoring board served as a platform for each trial group to share their progress and the challenges of recruitment. These not only allowed each trial group to have access to outside expertise on recruitment but also created a timely monitoring and feedback system. However, these interactions may have reduced the independence of each trial group’s response to the perceived effective strategies and barriers.

Several key strategies might have contributed to our success in recruiting a relatively large number of low-income racial/ethnical minority families from various settings, including clinics, community settings, and schools. We learned that careful planning throughout the recruitment process and a sufficient budget for recruitment are critical for recruitment success. Having a large enough budget provides a financial basis for providing incentives, such as monetary incentives, transportation reimbursement, and childcare to compensate for the time and effort of participating in assessments. Personnel-related strategies include using trusting community partnerships and hiring recruitment staff from the community who are personality appropriate and willing to work flexible hours. Investigators should develop clear and detailed protocols for contacting participants, actively approach potential participants, recruit at times and locations that are convenient for participants, evaluate whether participants understand the activities and expectations of the study, assess participation motivation, and enroll only those who plan to continue participating over the study period and who have provided sufficient baseline data. It also proved helpful to use a tracking database and to promise to return clinical assessment results to participants.

## Conclusions

The recruitment of low-income racial/ethnic minority families from wide-ranging geographic areas and diverse settings into obesity prevention and treatment trials can be accomplished using comprehensive strategies that include: (1) community partnership support, (2) hiring recruitment staff who are culturally sensitive, personality appropriate, and willing to work flexible hours, and (3) repeated contacts with potential study participants.

## Additional files

Additional file 1: **Table S1.** Open-ended questions used to ask each trial group about recruitment strategies they had used. (DOCX 25 kb)

Additional file 2: **Figure S1.** Visual aids used by Minnesota at the consent process to help potential participants understand the activities of the trial. (PDF 120 kb)

Additional file 3: **Figure S2.** Visual aids used by Minnesota at the consent process to help potential participants understand the data collection visits and incentives. (PDF 147 kb)

Additional file 4: **Table S2.** Reasons for ineligibility, no consent, and no randomization. (DOCX 19 kb)

Additional file 5: **Table S3.** Baseline characteristics of COPTR participants and their caregivers. (DOCX 21 kb)

## Data Availability

The data for this article are presented in the tables and figures.

## Abbreviations

BMI: Body mass index
COPTR consortium: Childhood Obesity Prevention and Treatment Research consortium
CWRU: Case Western Reserve University
NIH: National Institutes of Health
RCRAE: Recruitment, consent, retention and adverse events
RCU: Research coordinating unit
