# Supplementary material for: Recruitment strategies for predominantly low-income, multi-racial/ethnic children and parents to 3-year community-based intervention trials: Childhood Obesity Prevention and Treatment Research (COPTR) Consortium
Source: Trials. 2019 May 28;20:296. doi: 10.1186/s13063-019-3418-0 (PMC6540365; doi:10.1186/s13063-019-3418-0)
Supplement: Supplementary file 1 — Table S1. Open-ended questions used to ask each trial group about recruitment strategies they had used. (DOCX 25 kb) [file 13063_2019_3418_MOESM1_ESM.docx]

**Additional Table S1.** Recruitment strategies employed in the COPTR study

| **Recruitment** | **Response** |
| --- | --- |
| **PLANNING** |  |
| Set recruitment goal |  |
| Define recruitment period |  |
| Define recruitment setting |  |
| Adequate budget for recruitment |  |
| Knowledge of the target population |  |
| Pilot test recruitment strategy |  |
| Identify community research collaborator(s) |  |
| Establish project communication strategies |  |
| Others, please specify |  |
| **RECRUITMENT STAFFING** |  |
| Culture sensitive (e.g., staff who represent target population) |  |
| Number of staff by full time/part time and language | __ FT English only  __ PT English only  __ FT English & Spanish  __ PT English & Spanish  __ FT other language  __ PT other language |
| Establish strong community links |  |
| Written training protocol |  |
| Others, please specify |  |
| **COMMUNITY OUTREACH AND PARTICIPANT IDENTIFICATION** |  |
| Community outreach by type (e.g., church, school, or club events) |  |
| Interest meeting for potential participants |  |
| Advertisement by media of type and language |  |
| Personalized recruitment letter |  |
| Word-of-mouth referral |  |
| On-site recruitment |  |
| Collaborators’ referral |  |
| Others, please specify |  |
| **ELIGIBILITY SCREENING** |  |
| Active follow-up of potential participants |  |
| A protocol for maximum times of phone and mail contacts |  |
| Prioritized recruitment efforts |  |
| Recruitment tracking database |  |
| Participants' convenience |  |
| **CONSENT AND ASSENT** |  |
| Make consent process easy to understand |  |
| Assess participants' motivation for research study |  |
| Ensure participants understand benefits and potential risk |  |
| Clearly communicate the study procedure and staff's expectation |  |
| Introduce the importance of a control condition, random assignment and attrition bias to participants |  |
| Emphasize the importance of participation to others |  |
| Assure participants of the privacy and confidentiality of their information |  |
| Provide initial test results to participants |  |
| Strategies used to verify participant understand the study's requirements |  |
| A letter/instruction following screening |  |
| Others, please specify |  |
| **MEASUREMENT, INCENTIVES, AND ELIGIBILITY** |  |
| Place to conduct the measurement |  |
| Incentive structure |  |
| Reimburse transportation cost |  |
| Not recruit those repeatedly no show for data collection or hard to contact |  |
| Provide childcare and/or child activities |  |
| Others, please specify |  |
