# Supplementary material for: Recruitment strategies for predominantly low-income, multi-racial/ethnic children and parents to 3-year community-based intervention trials: Childhood Obesity Prevention and Treatment Research (COPTR) Consortium
Source: Trials. 2019 May 28;20:296. doi: 10.1186/s13063-019-3418-0 (PMC6540365; doi:10.1186/s13063-019-3418-0)
Supplement: Supplementary file 2 — Figure S1. Visual aids used by Minnesota at the consent process to help potential participants understand the activities of the trial. (PDF 120 kb) [file 13063_2019_3418_MOESM2_ESM.pdf]

NET-WORKS
NOW EVERYBODY TOGETHER FOR AMAZING AND HEALTHY KIDS

500 Families

"Measure-Me" Visits
& &
Up to $50 in Gift Cards

Year-1 "Measure-Me" Visits
& &
Up to $50 in Gift Cards

Year-2 "Measure-Me" Visits
& &
Up to $50 in Gift Cards

Year-3 "Measure-Me" Visits
& &
Up to $50 in Gift Cards

250 Families

NET-Works Group
3 Years

Information @ Well-Child Visits

Parenting Classes

Home Visits & Phone Calls

250 Families

Comparison Group
3 Years

Information @ Well-Child Visits

School Readiness Mailings

Contact Us: (612) 624-9105 or NET-Works@umn.edu
