# Supplementary material for: Recruitment strategies for predominantly low-income, multi-racial/ethnic children and parents to 3-year community-based intervention trials: Childhood Obesity Prevention and Treatment Research (COPTR) Consortium
Source: Trials. 2019 May 28;20:296. doi: 10.1186/s13063-019-3418-0 (PMC6540365; doi:10.1186/s13063-019-3418-0)
Supplement: Supplementary file 3 — Figure S2. Visual aids used by Minnesota at the consent process to help potential participants understand the data collection visits and incentives. (PDF 147 kb) [file 13063_2019_3418_MOESM3_ESM.pdf]

"Measure-Me" Visits

Visit 1

A. Eligibility and Consent
B. Height & Weight
C. Survey
D. Food Recall
E. Activity Monitor
F. Child Vocabulary
G. Hair Sample
H. Home Food Inventory
I. $10 Gift Card

Visit 2

A. Survey
B. Food Recall
C. Activity Monitor
D. Child Vocabulary
E. $10 Gift Card

Visit/Call 3

A. Food Recall
B. $30 Gift Card by mail

Contact Us: 612-624-9105 or NET-Works@umn.edu
