# Supplementary material for: Recruitment strategies for predominantly low-income, multi-racial/ethnic children and parents to 3-year community-based intervention trials: Childhood Obesity Prevention and Treatment Research (COPTR) Consortium
Source: Trials. 2019 May 28;20:296. doi: 10.1186/s13063-019-3418-0 (PMC6540365; doi:10.1186/s13063-019-3418-0)
Supplement: Supplementary file 4 — Table S2. Reasons for ineligibility, no consent, and no randomization. (DOCX 19 kb) [file 13063_2019_3418_MOESM4_ESM.docx]

**Additional Table S2.** Reasons for ineligibility, no consent, and no randomization

|  | **Minnesota** | |  | **Vanderbilt^*^** | |  | **Stanford** | |  | **CWRU** | |
| --- | --- | --- | --- | --- | --- | --- | --- | --- | --- | --- | --- |
| **Ineligibility Reasons** | **(n=1792)** | |  | **(n=291)** | |  | **(n=178)** | |  | **(n=442)** | |
| Child’s Age | 9 | 0.50% |  | 12 | 4.10% |  | 5 | 2.80% |  | 0 | 0.00% |
| Child’s BMI | 149 | 8.30% |  | 73 | 25.10% |  | 41 | 23.00% |  | 0 | 0.00% |
| Moved/Plan to move | 161 | 9.00% |  | 78 | 26.80% |  | 29 | 16.30% |  | 3 | 0.70% |
| Medical Condition/Medication | 13 | 0.70% |  | 20 | 6.90% |  | 12 | 6.70% |  | 15 | 3.40% |
| Language/income/other | 306 | 17.10% |  | 54 | 18.60% |  | 7 | 3.90% |  | 7 | 1.60% |
| No Qualifying Home/ Caregiver | 1 | 0.10% |  | 9 | 3.10% |  | 29 | 16.30% |  | 0 | 0.00% |
| Not interested | 1002 | 55.90% |  | 45 | 15.50% |  | 52 | 29.20% |  | 417 | 94.30% |
| PI Withdrawal | 150 | 8.40% |  | 0 | 0.00% |  | 3 | 1.70% |  | 0 | 0.00% |
| **Reasons for No Consent** | **(n=30)** | |  | **(n=996)** | |  | **(n=155)** | |  | **(n=286)** | |
| No longer interested | 23 | 76.70% |  | 297 | 29.80% |  | 92 | 59.40% |  | 26 | 9.10% |
| Unable to contact | 0 | 0.00% |  | 136 | 13.70% |  | 29 | 18.70% |  | 190 | 66.40% |
| Unable to schedule | 0 | 0.00% |  | 290 | 29.10% |  | 23 | 14.80% |  | 69 | 24.10% |
| Ineligible post- screening | 0 | 0.00% |  | 273 | 27.40% |  | 11 | 7.10% |  | 1 | 0.30% |
| Investigator withdrawal | 7 | 23.30% |  | 0 | 0.00% |  | 0 | 0.00% |  | 0 | 0.00% |
| **Reasons Not Randomized** | **(n=107)** | |  | **(n=229)** | |  | **(n=31)** | |  | **(n=32)** | |
| Child BMI | 0 | 0.00% |  | 0 | 0.00% |  | 3 | 9.70% |  | 18 | 56.30% |
| No longer Interested | 16 | 15.00% |  | 76 | 33.20% |  | 7 | 22.60% |  | 0 | 0.00% |
| Unable to contact | 1 | 0.90% |  | 36 | 15.70% |  | 0 | 0.00% |  | 0 | 0.00% |
| Unable to schedule | 0 | 0.00% |  | 24 | 10.50% |  | 0 | 0.00% |  | 0 | 0.00% |
| Incomplete baseline | 77 | 72.00% |  | 92 | 40.20% |  | 16 | 51.60% |  | 11 | 34.40% |
| Ineligible post-consent | 0 | 0.00% |  | 0 | 0.00% |  | 4 | 12.90% |  | 3 | 9.40% |
| Investigator withdrawal | 13 | 12.10% |  | 0 | 0.00% |  | 1 | 3.20% |  | 0 | 0.00% |

**^*^** Vanderbilt had a two-step eligibility assessment process where interest was assessed and then at a separate location BMI eligibility was assessed prior to consent.
