# Supplementary material for: Recruitment strategies for predominantly low-income, multi-racial/ethnic children and parents to 3-year community-based intervention trials: Childhood Obesity Prevention and Treatment Research (COPTR) Consortium
Source: Trials. 2019 May 28;20:296. doi: 10.1186/s13063-019-3418-0 (PMC6540365; doi:10.1186/s13063-019-3418-0)
Supplement: Supplementary file 5 — Table S3. Baseline characteristics of COPTR participants and their caregivers. (DOCX 21 kb) [file 13063_2019_3418_MOESM5_ESM.docx]

**Additional Table S3.** Baseline characteristics of COPTR participants and their caregivers

|  | **Minnesota** | |  | **Vanderbilt** | |  | **Stanford** | |  | **CWRU** | |
| --- | --- | --- | --- | --- | --- | --- | --- | --- | --- | --- | --- |
| **Characteristics** | **(N = 534)** | |  | **(N = 610)** | |  | **(N = 241)** | |  | **(N = 360)** | |
|  | % or mean | SD |  | % or mean | SD |  | % or mean | SD |  | % or mean | SD |
| **Children** |  |  |  |  |  |  |  |  |  |  |  |
| Age (years) | 3.4 | 0.7 |  | 4.3 | 0.9 |  | 9.5 | 1.4 |  | 11.6 | 0.6 |
| Gender (% male) | 49.1 |  |  | 48.2 |  |  | 44.5 |  |  | 42.2 |  |
| Race/ethnicity (%) |  |  |  |  |  |  |  |  |  |  |  |
| Non-Hispanic White | 12.4 |  |  | 1.0 |  |  | 0 |  |  | 3.9 |  |
| Non-Hispanic African American | 18.2 |  |  | 5.9 |  |  | 1.7 |  |  | 76.7 |  |
| Hispanic | 57.9 |  |  | 90.0 |  |  | 97.5 |  |  | 16.4 |  |
| Multi-racial | 6.7 |  |  | 0.5 |  |  | 0.4 |  |  | 2.2 |  |
| Other | 4.9 |  |  | 2.6 |  |  | 0.4 |  |  | 0.8 |  |
| BMI Percentile | 81.7 | 14.3 |  | 77.1 | 13.0 |  | 96.5 | 3.2 |  | 95.7 |  |
| BMI categories (%) |  |  |  |  |  |  |  |  |  |  |  |
| 50^th^ - <85^th^ percentile | 51.7 |  |  | 65.2 |  |  | 0 |  |  | 0 |  |
| ≥85^th^ - <95^th^ percentile | 25.7 |  |  | 33.9 |  |  | 24.1 |  |  | 32.5 |  |
| ≥95^th^ percentile | 22.7 |  |  | 0.8 |  |  | 75.9 |  |  | 67.5 |  |
| **Caregivers** |  |  |  |  |  |  |  |  |  |  |  |
| Age (years) | 31.9 | 6.4 |  | 32.2 | 6.7 |  | 36.7 | 6.9 |  | 38.2 | 8.1 |
| Marital status (% single) | 31.3 |  |  | 17.1 |  |  | 14.1 |  |  | 66.7 |  |
| Born outside US (% yes) | 62.0 |  |  | 89.1 |  |  | 93.8 |  |  | - |  |
| Highest household education (%) |  |  |  |  |  |  |  |  |  |  |  |
| <High school graduate | 33.3 |  |  | 51.9 |  |  | 63.5 |  |  | 20.5 |  |
| High school graduate | 22.1 |  |  | 25.8 |  |  | 17.0 |  |  | 27.5 |  |
| At least some college | 44.5 |  |  | 22.4 |  |  | 19.5 |  |  | 52.0 |  |
| Household Income (%) |  |  |  |  |  |  |  |  |  |  |  |
| <$35,000 | 80.9 |  |  | 69.2 |  |  | 55.2 |  |  | 62.0 |  |
| ≥$35,000 - < $50,000 | 9.9 |  |  | 2.6 |  |  | 13.3 |  |  | 10.0 |  |
| ≥$50,000 | 9.2 |  |  | 0.7 |  |  | 6.7 |  |  | 9.7 |  |
| Don’t know or no answer | 0 |  |  | 27.5 |  |  | 24.9 |  |  | 18.3 |  |
| SNAP participant (% yes) | 43.0 |  |  | 75.3 |  |  | 40.7 |  |  | 70.6 |  |
| Food Security (%) |  |  |  |  |  |  |  |  |  |  |  |
| High or marginal | 62.6 |  |  | 57.8 |  |  | 57.7 |  |  | 64.6 |  |
| Low | 31.2 |  |  | 28.2 |  |  | 31.1 |  |  | 23.7 |  |
| Very low | 6.3 |  |  | 13.9 |  |  | 11.2 |  |  | 11.7 |  |
| BMI (kg/m^2^) | 29.8 | 6.7 |  | 29.6 | 5.8 |  | 31.8 | 6.4 |  | 35.7 | 8.9 |
